# A GPS Phase-Locked Loop Performance Metric Based on the Phase Discriminator Output

**DOI:** 10.3390/s18010296

**Published:** 2018-01-19

**Authors:** Stefan Stevanovic, Boris Pervan

**Affiliations:** Illinois Institute of Technology (IIT), Chicago, IL 60616, USA; pervan@iit.edu

**Keywords:** navigation, GNSS, GPS, PLL, carrier tracking, tracking error, interference, wideband interference, oscillator, phase-noise

## Abstract

We propose a novel GPS phase-lock loop (PLL) performance metric based on the standard deviation of tracking error (defined as the discriminator’s estimate of the true phase error), and explain its advantages over the popular phase jitter metric using theory, numerical simulation, and experimental results. We derive an augmented GPS phase-lock loop (PLL) linear model, which includes the effect of coherent averaging, to be used in conjunction with this proposed metric. The augmented linear model allows more accurate calculation of tracking error standard deviation in the presence of additive white Gaussian noise (AWGN) as compared to traditional linear models. The standard deviation of tracking error, with a threshold corresponding to half of the arctangent discriminator pull-in region, is shown to be a more reliable/robust measure of PLL performance under interference conditions than the phase jitter metric. In addition, the augmented linear model is shown to be valid up until this threshold, which facilitates efficient performance prediction, so that time-consuming direct simulations and costly experimental testing can be reserved for PLL designs that are much more likely to be successful. The effect of varying receiver reference oscillator quality on the tracking error metric is also considered.

## 1. Introduction

This work describes a technique to evaluate the performance GPS receivers that track carrier phase and are vulnerable to wideband radio frequency interference (RFI). The approach is valid for any GPS application in which the receiver cannot tolerate cycle slips in the phase-lock loop (PLL). The method is directly applicable to ground-based reference receivers for differential GPS systems, as well as other ground-based receivers that require high continuity of service. It is also relevant to roving receivers, if the additional dynamic stresses on the PLL are also taken into account.

The example application motivating this work is Ground Based Augmentation System (GBAS) reference station receivers subjected to broadband interference, for example, from nearby use of personal privacy devices (PPDs). Prior work [1,2] has shown that PPDs most commonly emit broadband interference, and GBAS ground based reference receivers have experienced tracking discontinuities as a result [3]. These events can cause navigation service interruptions to aircraft on final approach. To ensure continuity of the navigation service, GBAS reference stations must be able to track GPS signals in the presence of wideband interference.

A popular metric used to predict the lock performance of a PLL under noisy conditions is the total *phase jitter* metric [4,5,6,7]. A major advantage of the jitter metric over more rigorous/accurate performance evaluation tools, such as computing the mean time to cycle slip (MTCS), is in its analytical simplicity. A closed form expression for the MTCS has only been derived assuming a simple first-order phase-lock loop with additive white Gaussian noise (AWGN) [8]. Higher-order loops are much more difficult to analyze, and, in such cases, the MTCS would need to be determined via time-consuming direct PLL simulation [9]. Additional considerations such as oscillator phase-noise and dynamic effects are not currently within the capability of the non-linear theory. In contrast, the phase jitter metric (using linear theory) is able to account for oscillator phase-noise and dynamics. However, the phase jitter metric is not without drawbacks. Regardless of whether a linear or non-linear model is used in theoretical analysis, simulation and/or experimental results are required in practice to validate PLL designs.

Prior research [5] relying on the jitter metric has capitalized on the idea that reducing the noise-equivalent bandwidth of the phase-lock loop will reduce the impact of wideband interference. The jitter metric was used as a design tool to determine the minimum PLL bandwidth and carrier-to-noise ratio ($C/N_{0}$) at which a GPS PLL would track reliably. However, experimental results in [5] showed that the PLL was cycle slipping (and losing lock) at higher carrier-to-noise ratios than predicted by the phase jitter metric. In fact, results from other work [4,6,7] consistently state that in experiments, loss of lock occurs at $C/N_{0}$ values that are higher than predicted by the phase jitter metric. In [6], analysis relying on the phase jitter metric led the authors to claim that increasing coherent averaging time would not improve PLL tracking capability in the presence of wideband interference. However, we will show that increasing coherent averaging time is essential for maintaining lock through interference events. Furthermore, our conclusions will be supported by theory as well as experimental results.

In this work, we propose the standard deviation of *tracking error* (defined here as the discriminator’s estimate of the true phase error) be used as a PLL performance design metric and expound the advantages of this metric over the popular phase jitter metric (which is based on the true phase error) using theory, numerical simulation, and experimental results. We derive an augmented GPS phase-lock loop (PLL) linear model, which includes the effect of coherent averaging, to be used in conjunction with this proposed metric. This model is functionally similar to those in [10,11]. However, the focus of that existing work was on novel discriminator and loop filter designs, while our paper focuses on a PLL design and *performance analysis tool* based on the tracking error.

The augmented linear model allows for more accurate calculation of tracking error standard deviation in the presence of AWGN as compared to the traditional linear model. The standard deviation of tracking error, with a threshold corresponding to half of the arctangent discriminator pull-in region, is shown to be a more reliable/robust measure of PLL performance under interference conditions than the phase jitter metric. In addition, the augmented linear model is shown to be valid up until this threshold, which facilitates efficient performance prediction, thus time-consuming direct simulations and costly experimental testing can be reserved for designs that are much more likely to be successful. The effect of varying receiver reference oscillator quality on the tracking error metric is also considered.

Section 2 briefly explains GPS PLL operation, and the augmented linear model is derived in Section 3 from first principles. The effects of AWGN and reference oscillator phase-noise are quantified. Section 4 explains the clock phase-noise models, and the popular phase jitter metric is defined in Section 5. In Section 6, we gain some insight into the effects of PLL bandwidth and coherent averaging time on the phase jitter and tracking error metrics. The effect of AWGN on the augmented linear model is validated in Section 7, and the new PLL performance metric based on the tracking error is explained in Section 8. Insight is gained into which performance improvement techniques will be the most effective at preventing PLL cycle slips in the presence of wideband interference. Tightening the PLL bandwidth is shown to be of no benefit in rejecting noise on the tracking error, while increasing the coherent averaging time provides the most benefit. Finally, the effect of oscillator phase-noise modeling on the tracking error metric is discussed in Section 9, and the theory developed in this paper is compared with experimental results.

## 2. PLL Operation

**Figure 1:**
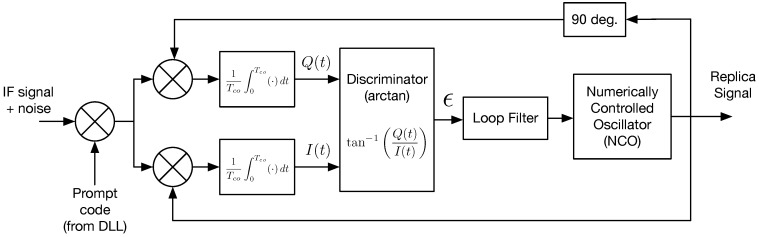
Phase lock loop block diagram.

The phase lock loop (PLL) is essentially a feedback control system that creates a replica carrier signal and attempts to keep its frequency and phase aligned with the incoming carrier signal. This work focuses specifically on the PLL because it is most susceptible to wideband interference, and will lose lock before the delay-lock loop (DLL) does [5]. Figure 1 shows the PLL block diagram. The input to the PLL is the intermediate frequency (IF) signal s(t), which may be written for a single satellite as,

(1) $$\begin{array}{cc} s(t) & =\sqrt{2C}D\left(t-\tau \right)x\left(t-\tau \right)\cos\left(\omega_{IF}t+\psi \left(t\right)\right) \end{array}$$

where *C* is the signal power, *D* is the navigation data message, *x* is the code, τ is the code delay, $\omega_{IF}$ is the intermediate frequency (in rad/s), and ψ(t) is the phase process which includes all Doppler effects due to user and satellite motion, as well as both receiver and satellite clock instabilities. The receiver clock instability is a consequence of the down-conversion in the receiver’s front-end.

The input signal is first multiplied by the code replica obtained from the receiver’s delay lock loop (DLL). This process is called code wipe-off because, if the replica code is aligned perfectly with the incoming, the code will be removed from the signal. In this work, perfect code alignment is assumed, which allows the signal, after mixing with prompt code, to be written as,

(2) $$\begin{array}{cc} y(t) & =\sqrt{2C}D\left(t-\tau \right)\cos\left(\omega_{IF}t+\psi \left(t\right)\right) \end{array}$$

The signal y(t) is then multiplied by two replica signals from the numerically controlled oscillator (NCO), called in-phase and quadrature signals, $x_{I}$ and $x_{Q}$, respectively,

(3) $$\begin{array}{cc} x_{I}\left(t\right) & =\sqrt{2}K\cos\left(\omega_{IF}t+\hat{\psi }\left(t\right)\right) \end{array}$$

(4) $$\begin{array}{cc} x_{Q}\left(t\right) & =-\sqrt{2}K\sin\left(\omega_{IF}t+\hat{\psi }\left(t\right)\right) \end{array}$$

where *K* is the RMS amplitude and $\hat{\psi }\left(t\right)$ is the replica phase process. This mixes the signal down to baseband (carrier wipe-off). Then there is a coherent averaging (i.e., integrate and dump) operation followed by the discriminator, which is sometimes called a phase detector because it estimates the phase error between input and replica signals. In this work, the discriminator output is called the tracking error. The loop filter is a compensator designed to achieve desired system response, and it designates the ‘order’ of the phase lock loop [12]. The loop filter output is fed to the numerically controlled oscillator (NCO) which generates the replica carrier signals, and closes the loop. Additional detailed information on the complete GPS signal processing technique (from signal capture at the antenna through pseudorange computation) is available in [12,13,14].

The receiver clock is a reference input to the NCO. Therefore, the receiver clock phase-noise can be modeled as a disturbance on the NCO output. However, in this work, the PLL is implemented in software, and there is no additional phase-noise contribution from the NCO. The effect of both satellite and receiver oscillator phase noise will enter the PLL through the input signal (due to mixing the the receiver front-end). In addition, our PLL uses the common two-quadrant arctangent discriminator and a third-order loop filter (more on the loop filter in Section 3).

## 3. Augmented PLL Linear Model

In this section, we derive a linear model for the PLL shown in Figure 1. The advantage of a linear model is that it may be analyzed using conventional control system techniques. The derivation that follows assumes “small” levels of additive white Gaussian noise (AWGN), or “high” $C/N_{0}$. We anticipate that the model will likely break down for low carrier-to-noise ratios we are interested in. However, in Section 8, we will show that the linear model produces accurate results at much lower carrier-to-noise ratios than expected.

The PLL input signal y(t), following code wipe-off, may be written as,

(5) $$\begin{array}{cc} y(t) & =\sqrt{2}A\cos\left(\omega_{IF}t+\psi \left(t\right)\right)+n\left(t\right) \end{array}$$

where *A* is the RMS amplitude, and n(t) is assumed AWGN (it is actually band-passed in the front-end, but the PLL bandwidth is much smaller, so we will assume AWGN for simplicity). The navigation data D(t−τ) are not included in Equation (5) because we are assuming the data bit value does not change during a coherent averaging period. This can be ensured by using coherent averaging times of less than the duration of one bit.

Note that we can also write the additive noise as,

(6) $$\begin{array}{c} n\left(t\right)=\sqrt{2}n_{I}\left(t\right)\cos\left(\omega_{IF}t\right)-\sqrt{2}n_{Q}\left(t\right)\sin\left(\omega_{IF}t\right) \end{array}$$

where $n_{I}\left(t\right)$ and $n_{Q}\left(t\right)$ are independent WGN processes ([15], p. 64).

The in-phase signal after mixing with replica from Equation (3) may be written as,

(7) $$\begin{array}{cc} I(t) & =y\left(t\right)x_{I}\left(t\right)\\ & =2AK\cos\left(\omega_{IF}t+\psi \left(t\right)\right)\cos\left(\omega_{IF}t+\hat{\psi }\left(t\right)\right) \end{array}$$

(8) $$\begin{array}{c} \;+n\left(t\right)\sqrt{2}K\cos\left(\omega_{IF}t+\hat{\psi }\left(t\right)\right) \end{array}$$

Substituting Equation (6) for n(t), and using product-to-sum trigonometric identities, we have,

(9) $$\begin{array}{cc} I(t) & =AK\cos(\psi \left(t\right)-\hat{\psi }\left(t\right))\\ & \;+K[n_{I}\left(t\right)\cos\hat{\psi }\left(t\right)+n_{Q}\left(t\right)\sin\hat{\psi }\left(t\right)] \end{array}$$

where the resultant higher-frequency components are neglected. They will be filtered out by coherent averaging, which follows the mixing operation. Finally, defining the phase error as $\phi \left(t\right):=\psi \left(t\right)-\hat{\psi }\left(t\right)$, the in-phase signal is,

(10) $$\begin{array}{cc} I(t) & =AK\left[\cos\phi \left(t\right)+n_{I}^{\prime }\left(t\right)\right] \end{array}$$

with,

(11) $$\begin{array}{cc} n_{I}^{\prime }\left(t\right) & :=\frac{1}{A}\left[n_{I}\left(t\right)\cos\hat{\psi }\left(t\right)+n_{Q}\left(t\right)\sin\hat{\psi }\left(t\right)\right] \end{array}$$

Similarly, we can write the quadrature signal as,

(12) $$\begin{array}{cc} Q(t) & =y\left(t\right)x_{Q}\left(t\right)\\ & =AK\sin(\psi \left(t\right)-\hat{\psi }\left(t\right)) \end{array}$$

(13) $$\begin{array}{c} \;+K[n_{Q}\left(t\right)\cos\hat{\psi }\left(t\right)-n_{I}\left(t\right)\sin\hat{\psi }\left(t\right)] \end{array}$$

(14) $$\begin{array}{cc} Q(t) & =AK\left[\sin\phi \left(t\right)+n_{Q}^{\prime }\left(t\right)\right] \end{array}$$

with,

(15) $$\begin{array}{cc} n_{Q}^{\prime }\left(t\right) & :=\frac{1}{A}\left[n_{Q}\left(t\right)\cos\hat{\psi }\left(t\right)-n_{I}\left(t\right)\sin\hat{\psi }\left(t\right)\right] \end{array}$$

The IQ signals are then filtered in the coherent averaging operation (denoted using angled brackets), and the tracking error is computed as,

(16) $$\begin{array}{c} \epsilon \left(t\right):=\tan^{-1}\left(\frac{\langle Q(t)\rangle }{\langle I(t)\rangle }\right) \end{array}$$

Substituting Equations (10) and (14) for I(t) and Q(t), respectively,

(17) $$\begin{array}{c} \epsilon \left(t\right)=\tan^{-1}\left(\frac{\langle \sin\phi \left(t\right)+n_{Q}^{\prime }\left(t\right)\rangle }{\langle \cos\phi \left(t\right)+n_{I}^{\prime }\left(t\right)\rangle }\right) \end{array}$$

To proceed further, we consider a large carrier-to-noise ratio, $C/N_{0}$, which implies that the additive noise is small. Under this condition, it is assumed that the PLL will be tracking properly, and therefore the phase error, ϕ(t), will be small. Linearizing the sine and cosine terms, we may write Equation (17) as,

(18) $$\begin{array}{c} \epsilon \left(t\right)=\tan^{-1}\left(\frac{\langle \phi \left(t\right)+n_{Q}^{\prime }\left(t\right)\rangle }{\langle 1+n_{I}^{\prime }\left(t\right)\rangle }\right) \end{array}$$

Now, the arctangent is linearized, and we focus on the denominator. First we note that the averaging applies only to the in-phase noise component. In addition, the binomial theorem can be used because the noise is assumed to be small.

(19) $$\begin{array}{ll} \epsilon (t) & =\frac{\langle \phi \left(t\right)+n_{Q}^{\prime }\left(t\right)\rangle }{1+\langle n_{I}^{\prime }\left(t\right)\rangle } \end{array}$$

(20) $$\;\begin{array}{l} \approx \langle \phi \left(t\right)+n_{Q}^{\prime }\left(t\right)\rangle \left(1-\langle n_{I}^{\prime }\left(t\right)\rangle \right) \end{array}$$

(21) $$\;\begin{array}{l} =\langle \left(\phi \left(t\right)+n_{Q}^{\prime }\left(t\right)\right)\left(1-\langle n_{I}^{\prime }\left(t\right)\rangle \right)\rangle \end{array}$$

(22) $$\;\begin{array}{l} =\langle \phi \left(t\right)+n_{Q}^{\prime }\left(t\right)-\phi \left(t\right)\langle n_{I}^{\prime }\left(t\right)\rangle -n_{Q}^{\prime }\left(t\right)\langle n_{I}^{\prime }\left(t\right)\rangle \rangle \end{array}$$

Since the phase error, ϕ, and additive noise is small, the 2nd-order small terms are neglected, resulting in,

(23) $$\begin{array}{cc} \epsilon (t) & =\langle \phi \left(t\right)+n_{Q}^{\prime }\left(t\right)\rangle \end{array}$$

**Figure 2:**
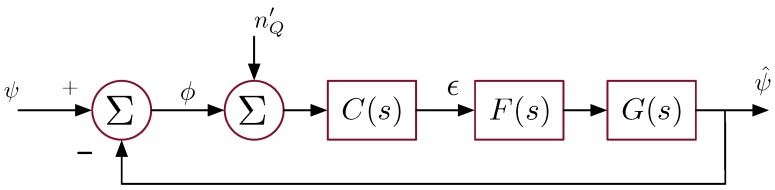
Augmented PLL linear model.

Figure 2 shows the linear block diagram drawn from Equation (23), where the transfer functions C(s), F(s), and G(s) correspond to the coherent integration, loop filter and NCO, respectively. This figure helps to clarify the definition of phase error, ϕ, and tracking error, ϵ, by visual representation.

The AWGN input is represented by $n_{Q}^{\prime }$, and since our PLL is implemented in software, there is no phase-noise contribution from the NCO. The phase-noise of both the receiver and satellite clocks will only enter the PLL through the input phase process, ψ, due to mixing in the receiver front-end. When working with traditional receivers, the added phase-noise contribution from the NCO can easily be included. Its absence in our particular PLL implementation does not affect the analysis techniques or conclusions reached using this linear model.

It is worth noting that the only difference between this augmented linear model and the conventional linear model ([9], pp. 127–128) is the additional effect of coherent averaging, C(s). It is included to obtain a more accurate transfer function from AWGN input to tracking error. We will see the impact this additional block has on transfer function computations in Section 6.

We ultimately want to express the variance of the tracking error, $\sigma_{\epsilon }^{2}$, by using the Wiener–Khinchin theorem. To do so, we need the transfer function from the noise and phase inputs to the tracking error, which we will call the “tracking error transfer function”.

Using C(s) to denote the transfer function for the coherent averaging, the tracking error transfer function, $H_{TE}$, is,

(24) $$\begin{array}{cc} H_{TE}\left(s\right) & =\frac{C(s)}{1+C(s)F(s)G(s)} \end{array}$$

This transfer function describes the following input–error relation,

(25) $$\begin{array}{cc} \epsilon (s) & =H_{TE}\left(s\right)\left(n_{Q}^{\prime }\left(s\right)+\psi \left(s\right)\right) \end{array}$$

Then, the variance of the *tracking error*, ϵ, can be computed as,

(26) $$\begin{array}{cc} \sigma_{\epsilon }^{2} & =\int_{0}^{\infty }{\left|H_{TE}\left(j2\pi f\right)\right|}^{2}\left(S_{n_{i}}\left(f\right)+S_{clk}\left(f\right)\right)\;df \end{array}$$

where $S_{n_{i}}\left(f\right)$ is the single-sided power spectral density (PSD) of the noise input, $n_{Q}^{\prime }$, and $S_{clk}\left(f\right)$ is the single-sided PSD of clock phase noise.

It can be shown that the relationship between the input noise PSD and carrier to noise ratio is,

(27) $$\begin{array}{cc} S_{n_{i}}\left(f\right) & =\frac{1}{\left(C/N_{0}\right)} \end{array}$$

when A=1 and K=1; these values are chosen for convenience in our simulations. The modeling of oscillator phase noise PSDs is discussed in Section 4.

In addition, we would like to write the variance of the *phase error*, $\sigma_{\phi }^{2}$, since this is the principal component of the phase jitter metric. The jitter metric will be formally introduced in Section 5. The variance of the phase error, ϕ, can be written as,

(28) $$\begin{array}{cc} \sigma_{\phi }^{2} & :=\sigma_{\phi ,n}^{2}+\sigma_{\phi ,\mathrm{clk}}^{2} \end{array}$$

where, $\sigma_{\phi ,n}^{2}$ represents the variance of ϕ due to AWGN, and $\sigma_{\phi ,\mathrm{clk}}^{2}$ represents the variance due to clock phase-noise. Using Figure 2, these variances may be written as,

(29) $$\begin{array}{cc} \sigma_{\phi ,n}^{2} & =\int_{0}^{\infty }{\left|H(j2\pi f)\right|}^{2}S_{n_{i}}\left(f\right)\;df \end{array}$$

(30) $$\begin{array}{cc} \sigma_{\phi ,\mathrm{clk}}^{2} & =\int_{0}^{\infty }{\left|1-H(j2\pi f)\right|}^{2}S_{clk}\left(f\right)\;df \end{array}$$

with the transfer function from the noise input to the output,

(31) $$\begin{array}{cc} H(s) & =\frac{C(s)F(s)G(s)}{1+C(s)F(s)G(s)} \end{array}$$

To evaluate H(s) and $H_{TE}\left(s\right)$, we must define C(s), F(s), and G(s).

For simplicity, the integrate and dump operation (i.e., coherent averaging) will be approximated using a moving average. The transfer function for a moving average, derived in Appendix B, can be expressed as,

(32) $$\begin{array}{cc} C(j\omega ) & =\frac{T}{T_{co}}\frac{1-e^{-j\omega T_{co}}}{1-e^{-j\omega T}} \end{array}$$

where, *T* is the sampling period and $T_{co}$ is the coherent integration time. The magnitude of C(jω) can be shown to be a sinc function,

(33) $$\begin{array}{c} {\left|C\left(j\omega \right)\right|}^{2}={\mathrm{sinc}}^{2}\left(\omega \frac{T_{co}}{2}\right) \end{array}$$

The loop filter (3rd-order) and NCO transfer functions are given by,

(34) $$\begin{array}{ll} F_{3}\left(j\omega \right) & =\frac{b_{3}w_{0,3}{\left(j\omega \right)}^{2}+a_{3}w_{0,3}^{2}\left(j\omega \right)+w_{0,3}^{3}}{{\left(j\omega \right)}^{2}} \end{array}$$

(35) $$\;\begin{array}{ll} G(j\omega ) & =\frac{1}{j\omega } \end{array}$$

A third-order loop filter is considered because of its superior ability to handle dynamics, and additional design freedom compared to a second-order loop [4]. It has zero steady-state error to acceleration stress. However, there will be a steady-state phase error for jerk stresses [12].

The loop filter coefficients $a_{3}$, $b_{3}$, and $w_{0,3}$ are chosen based on the design requirements. One important design consideration is the equivalent noise bandwidth, often simply referred to as the bandwidth. As shown in the following section, modifying the PLL bandwidth will alter the loop filter transfer function, F(s), to meet certain requirements based on noise performance.

### Noise-Equivalent Bandwidth

The noise-equivalent bandwidth is the “bandwidth of the ideal filter that would pass the same noise power as the filter under study”. The noise-equivalent bandwidth expressed in units of Hertz is [13],

(36) $$\begin{array}{c} B_{n}=\frac{1}{{2|H\left(0\right)|}^{2}}\int_{-\infty }^{\infty }{\left|H\left(j2\pi f\right)\right|}^{2}\;df \end{array}$$

where $B_{n}$ denotes a one-sided bandwidth, and subscript *n* means that it is the noise-equivalent bandwidth.

Equation (36) specifies the relationship between the bandwidth and loop filter coefficients. For example, a first-order loop has a single loop filter coefficient. Satisfying a bandwidth requirement would allow that single coefficient to be computed using Equation (36), thereby completing the loop filter design. Second and third order loop filters offer additional design freedom. For a third-order loop, two loop filter coefficients in addition to the bandwidth must be specified. The third-order loop filter coefficients used in this work are, $a_{3}=1.1$, $b_{3}=2.4$, and $w_{0,3}=0.7845B_{n}$, where, $a_{3}$ and $b_{3}$ are chosen to be the typical third-order loop filter coefficients specified in ([12], p. 180; Table 6.5). The only terms left to define in Equations (26) and (29) are the oscillator phase-noise PSDs.

## 4. Receiver Clock Comparison

The clock phase noise power spectral density (PSD), needed for satellite and receiver clocks in Equations (26) and (29), is modeled using a power law as [5,13],

(37) $$\begin{array}{c} S\left(f\right)=h_{0}f^{0}+h_{1}f^{-1}+h_{2}f^{-2}+h_{3}f^{-3}+h_{4}f^{-4} \end{array}$$

where S(f) is in units of dB/Hz, *f* is in Hz, and *h* coefficients for several common crystal and atomic oscillators are given in Table 1. Power law coefficents for the temperature compensated crystal oscillator (TCXO) and oven controlled crystal oscillator (OCXO) are taken from [13]. The rubidium model corresponds to the SpectraTime LPFRS-01/AV1 oscillator used when collecting experimental data, and the coefficient computation is detailed in Appendix A.

**Figure 3:**
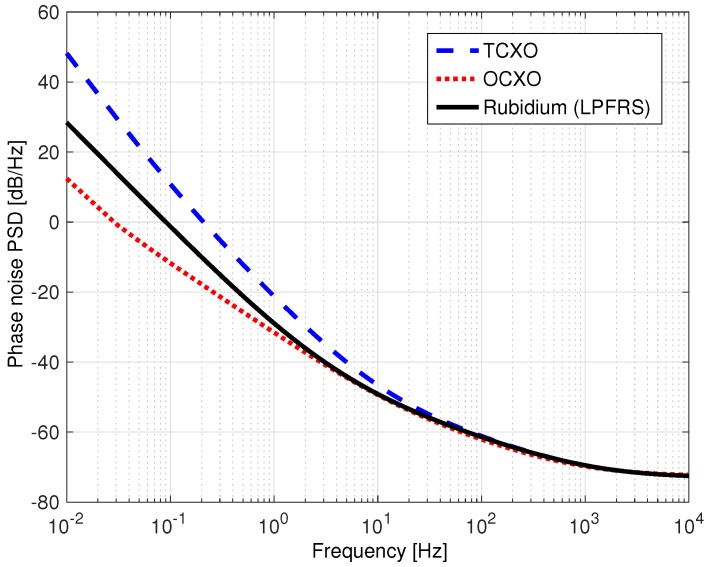
Receiver clock power spectral density (PSD) comparison.

The phase noise power spectral densities (PSDs) for these clocks are shown in Figure 3. Note that all PSDs coincide at high frequencies. This is because frequency synthesis is taken into account, and the synthesizer’s voltage controlled oscillator (VCO) dominates at higher frequencies. More information on phase noise in frequency synthesizers is available in [16,17]. While a rubidium clock remains stable (i.e., does not drift) over longer periods of time relative to a crystal oscillator, it does not necessarily have lower phase-noise. This is evident in Figure 3, where the PSD of our rubidium oscillator lies between the TCXO and OCXO. When choosing a reference oscillator, priority should be placed on frequency stability as well as low phase-noise to reduce its effect on the standard deviation of tracking error.

Prior work [6,18] has used very conservative methods to compute power law model *h* coefficients for space vehicle (SV) clocks. The authors essentially shifted the TXCO PSD curve such that it passes through a single point specified by the GPS Standard Positioning Service (SPS) document [19]. The result is a PSD curve that completely upper bounds that of the TCXO clock. This is extremely conservative considering that satellites are equipped with rubidium (or cesium) atomic oscillators [19]. Therefore, the authors divided the resulting PSD by a factor of 100 to reduce the conservatism. However, this would create a PSD which is lower than the TCXO/OCXO PSD for high frequencies. This is not a realistic PSD curve because, as previously discussed, high-frequency phase-noise is dominated by the VCO in the frequency synthesizer.

Since GPS satellites are equipped with either ceisium or rubidium atomic clocks, we assume that the SV clocks have a PSD representative of a rubidium atomic oscillator. In addition, in this work, we consider atomic clocks for use as receiver reference oscillators because they are becoming increasingly affordable and compact, and have high frequency stability compared to crystal oscillators. Even though this work focuses on stationary users, chip scale atomic clocks (CSACs) are available, and well suited for mobile navigation applications [4,5].

## 5. Phase Jitter Performance Metric

Up until this point, we have derived the linear model and defined PSDs for both AWGN and clock phase-noise. Now, we introduce the traditionally used jitter performance metric, and some issues in experimental results in prior work.

A commonly used test for analyzing tracking performance in the presence of dominant sources of error is often referred to as the total phase jitter rule. This rule states that the “three-sigma jitter must not exceed one-fourth of the phase pull-in range of the PLL discriminator” ([12], p. 184). For an inverse-tangent discriminator with an pull-in range of 180 degrees, this metric can be expressed (in units of degrees) as,

(38) $$\begin{array}{cc} 3\sigma_{PLL} & =3\sigma_{\phi }+\theta_{e}\leq 45\;\;\mathrm{degrees} \end{array}$$

where $\sigma_{PLL}$ is the total phase jitter, $\sigma_{\phi }$ is the standard deviation of phase error (from all sources, including clocks and thermal noise), and $\theta_{e}$ is the phase error caused by dynamic stresses. Since this work focuses on stationary receivers, dynamic stresses will be neglected.

Nominal contributions to phase jitter typically include satellite and receiver clock errors, and thermal noise [5,18]. The variance of phase error, $\sigma_{\phi }^{2}$, for a stationary receiver is defined in Equation (28). Again, note that this variance corresponds to the phase error, ϕ, in Figure 2.

The contribution due to additive white noise, $\sigma_{\phi ,n}^{2}$, derived in [20], can be expressed as,

(39) $$\begin{array}{cc} \sigma_{\phi ,n}^{2} & =\frac{B_{n}}{\left(C/N_{0}\right)}\left[1+\frac{1}{2T_{co}\left(C/N_{0}\right)}\right] \end{array}$$

where, $B_{n}$ is the single sided PLL bandwidth, $C/N_{0}$ is the carrier-to-noise ratio, and $T_{co}$ is the coherent integration time. Equation (39) would be equivalent to Equation (29), except for the second term in brackets (in Equation (39)) which accounts for squaring loss. In fact, by using Equations (27) and (36), Equation (29) can be reduced to the ratio of $B_{n}$ to $C/N_{0}$. Therefore, there is no squaring loss when an arctangent discriminator is used. This agrees with the result in [6].

The phase noise due to oscillator instabilities, $\sigma_{\phi ,\mathrm{clk}}^{2}$, is split into components due to satellite and receiver clocks as,

(40) $$\begin{array}{cc} \sigma_{\phi ,\mathrm{clk}}^{2} & =\sigma_{\phi ,n_{sv}}^{2}+\sigma_{\phi ,n_{rx}}^{2} \end{array}$$

with,

(41) $$\begin{array}{cc} \sigma_{\phi ,n_{sv}}^{2} & =\int_{0}^{\infty }{\left|1-H\left(j2\pi f\right)\right|}^{2}S_{sv}\left(f\right)\;df \end{array}$$

(42) $$\begin{array}{cc} \sigma_{\phi ,n_{rx}}^{2} & =\int_{0}^{\infty }{\left|1-H\left(j2\pi f\right)\right|}^{2}S_{rx}\left(f\right)\;df \end{array}$$

where $S_{sv}\left(f\right)$ and $S_{rx}\left(f\right)$ are the single-sided phase error PSDs for the satellite and receiver clocks, respectively [18].

The difference between the standard deviation of phase jitter and the standard deviation of tracking error (our proposed metric) can be seen in Figure 2. The phase jitter corresponds to the true phase error ϕ, while the tracking error, denoted as ϵ, is defined as the discriminator’s estimate of the phase error.

### Disadvantages of the Phase Jitter Metric

Experimental results from prior work [4,5,6,7] consistently show that in experiments, loss of lock occurs at $C/N_{0}$ values that are higher than predicted by the jitter metric. Based on analysis using the phase jitter metric, Razavi et al. [6] claim that “increasing coherent averaging time does not lower the *C*/*N* of the GPS signal which can be tracked if a tan-1 discriminator is used.” Later in the paper, we explain why increasing coherent averaging time does not affect the phase jitter. However, we show that increasing coherent averaging time does affect the tracking error, and we argue that the tracking error may in fact be a more reliable lock indicator metric for cycle slips. This new metric, used in conjunction with the augmented linear model, will help explain why increasing coherent averaging time is essential for maintaining lock in interference environments. Our conclusions are supported by ([12], p. 159) as well as our own theoretical and experimental results.

First, we will gain some insight into the augmented linear model, which will help us justify a new PLL design metric based on the PLL tracking error standard deviation. Then, we will compare this tracking error metric to the phase jitter metric and explain its advantages over the latter. We consider only the effect of AWGN for now. The effect of clock phase noise will be added later, in Section 9.

## 6. Bandwidth and Averaging Time Effects

This section explains how PLL bandwidth and coherent averaging time affect the magnitude of the input–output and tracking error transfer functions used in Equations (26) and (29).

**Figure 4:**
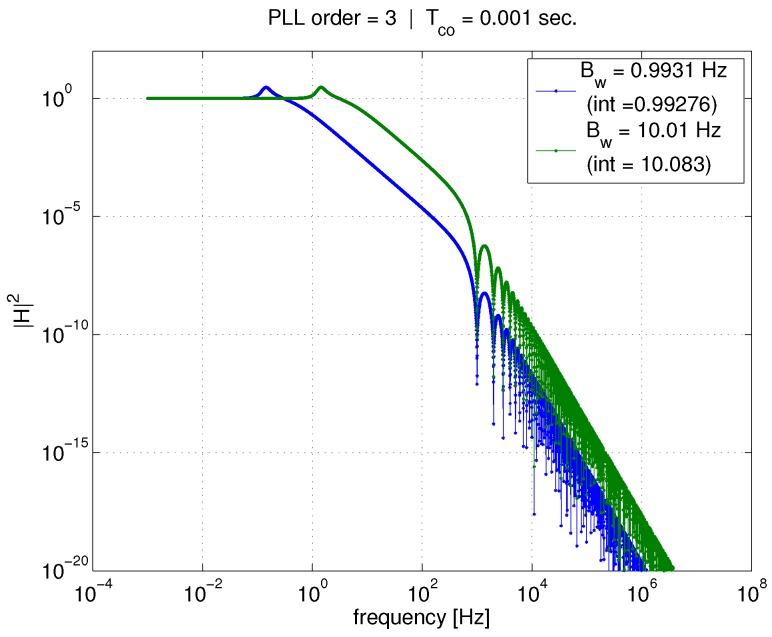
Effect of $T_{co}$ and PLL bandwidth on input–output transfer function, 1 ms.

**Figure 5:**
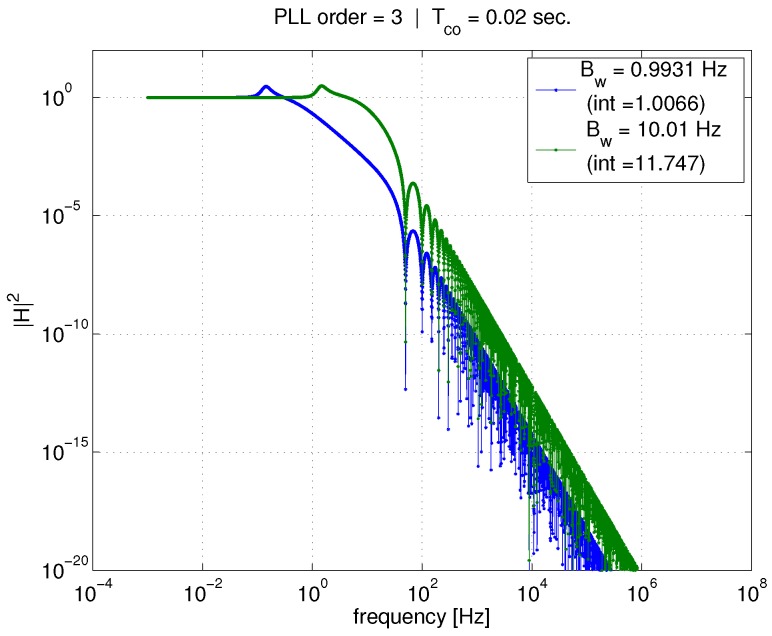
Effect of $T_{co}$ and PLL bandwidth on input–output transfer function, 20 ms.

Figure 4 shows the effect of PLL bandwidth on the *input–output* transfer function for a given coherent averaging time $T_{co}=1$ ms. The two curves represent PLL bandwidths of 1 and 10 Hz. At higher frequencies, the effect of coherent averaging (represented by the sinc function) is unmistakable, with the first lobe of the sinc function visible at about 1000 Hz. However, since the input–output transfer function is already a low-pass filter, there is little additional attenuation due to the coherent averaging (because the PLL noise bandwidth is much smaller than the bandwidth of the coherent averaging operation). In contrast, tightening the PLL bandwidth has a large impact on the transfer function magnitude curve, and results in a reduction in the integral of the transfer function (provided in the figure legend). Furthermore, note that Equation (29) relates the transfer function integral to the variance of phase error due to AWGN, $\sigma_{\phi ,n}^{2}$. Therefore, tightening PLL bandwidth will reduce $\sigma_{\phi ,n}^{2}$. Figure 5 shows the same curves, but corresponding to a 20 ms coherent averaging time. Again, a reduction in PLL bandwidth greatly affects the transfer function and its integral, and therefore the phase error variance, $\sigma_{\phi ,n}^{2}$.

Now, compare Figure 4 and Figure 5 to one another. For a given PLL bandwidth, increasing the coherent average time from 1 to 20 ms produces very little effect on the transfer function integral (again, given in figure legends) in comparison to the the effect of tightening bandwidth.

These figures illustrate the conclusions reached using the total phase jitter metric. When considering the effect of only AWGN, $\sigma_{\phi ,n}^{2}$ is the only component of, and thus equivalent to, the total phase jitter metric. Tightening PLL bandwidth should reduce the impact of AWGN on the phase error variance, thus improving the PLL’s ability to continuously track carrier phase. Another conclusion, which is stated in [6], is that increasing coherent averaging time would not help improve tracking ability/performance, since it does not significantly affect the phase error variance. While the latter is true, we will show in later sections of this paper that increasing coherent averaging time will definitely improve the PLL’s ability to continuously track carrier phase in the presence of AWGN.

**Figure 6:**
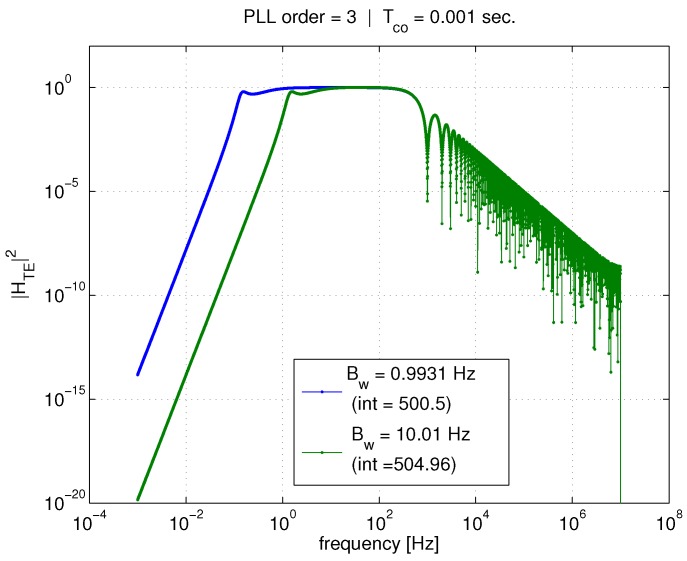
Effect of $T_{co}$ and PLL bandwidth on tracking error transfer function, 1 ms.

**Figure 7:**
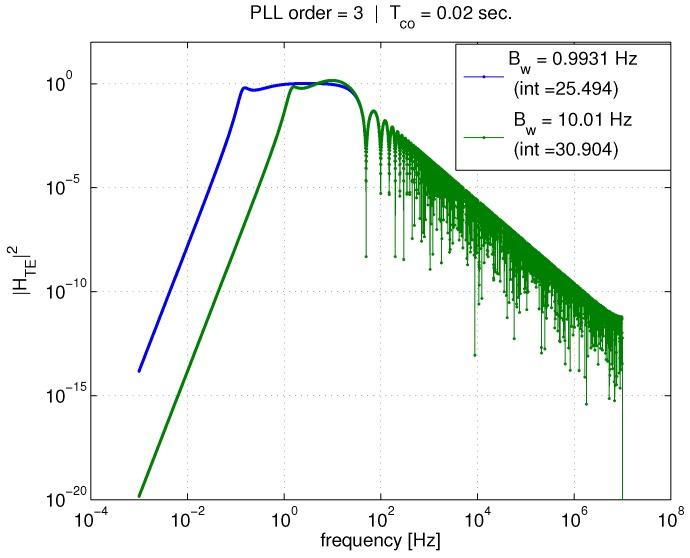
Effect of $T_{co}$ and PLL bandwidth on tracking error transfer function, 20 ms.

Now, we look at the same pair of figures, but for the *tracking error* transfer function. These are Figure 6 and Figure 7. The trends are reversed in this case. For a given PLL bandwidth, the averaging time significantly affects the transfer function integration, and thus the outcome of Equation (26). However, for a given $T_{co}$, reducing the bandwidth does not have a significant impact in comparison.

At higher frequencies, the coherent averaging is attenuating what would otherwise be a high-pass filter (if the standard linear model was used). Again, the first lobe of the sinc function is visible at about 1000 Hz in Figure 6. It is because of the addition of coherent averaging in the linear model that Equation (26) can now be used to compute a realistic value for the tracking error variance due to AWGN.

The next section shows these insights in graphical form, and validates that the tracking error variance is being accurately computed using the augmented linear model.

## 7. Validating Linear Model and Averaging Effects

In this section, we validate the augmented linear model tracking error calculation, and the effects of PLL bandwidth and coherent averaging time discussed in Section 6, using numerically simulated data. A simulation is used because we can isolate the effect of AWGN (and not have to worry about clock phase-noise).

An intermediate frequency (IF) signal with AWGN (similar to that which would be output from a receiver front-end) is numerically generated and used as the input to the software defined receiver (SDR). The generated IF is 5 MHz and is sampled at 20 MHz, which makes it identical to the experimental data which will be analyzed in Section 9; however, it does not contain the C/A code and navigation data. The SDR used to track the numerically generated signal is based on that provided with [14].

**Figure 8:**
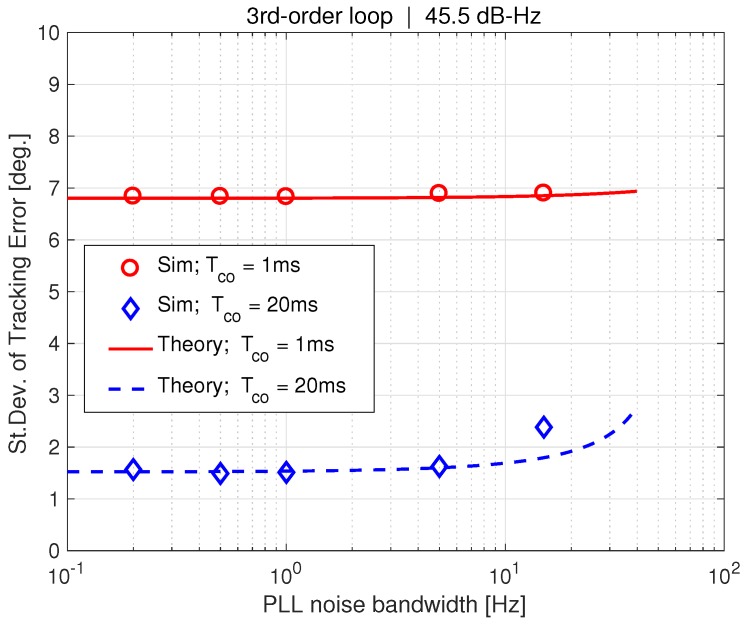
Sigma tracking error vs. PLL bandwidth.

Figure 8 shows the standard deviation of tracking error versus PLL bandwidth for both theory and simulation with a carrier-to-noise ($C/N_{0}$) ratio of 45.5 dB-Hz. A relatively high $C/N_{0}$ is used because the linear model was derived assuming small levels of noise. However, in the following section, it will be shown that the tracking error computation is valid for much smaller $C/N_{0}$, corresponding to interference conditions. Coherent average times of 1ms and 20ms are shown. Theory matches simulation very well at this relatively high $C/N_{0}$. However when $T_{co}=20$ ms, there is a slight deviation between the two as $\sigma_{\epsilon }$ begins to increase with increasing bandwidth. This increase in standard deviation has been noted in prior work [6], and it does not affect our theoretical conclusions since it is not caused by AWGN. It is caused by the PLL bandwidth approaching $\frac{1}{2T_{co}}$, the bandwidth of the coherent averaging, which is leading to loop stability issues. The results in Figure 8 clearly show that for a given $T_{co}$ the tracking error variance, $\sigma_{\epsilon }^{2}$, is not a function of the PLL bandwidth. However, increasing the coherent average time significantly reduces $\sigma_{\epsilon }^{2}$. Since only AWGN is considered, this shows the best performance possible, i.e., with a perfect oscillator.

Now, we can look at the standard deviation of the phase error, $\sigma_{\phi }$. The true phase error can easily be computed by differencing the PLL output from the simulated IF phase, which allows a cycle slip to be easily detected. A cycle slip occurs if the true phase error reaches one cycle, or 360 degrees.

**Figure 9:**
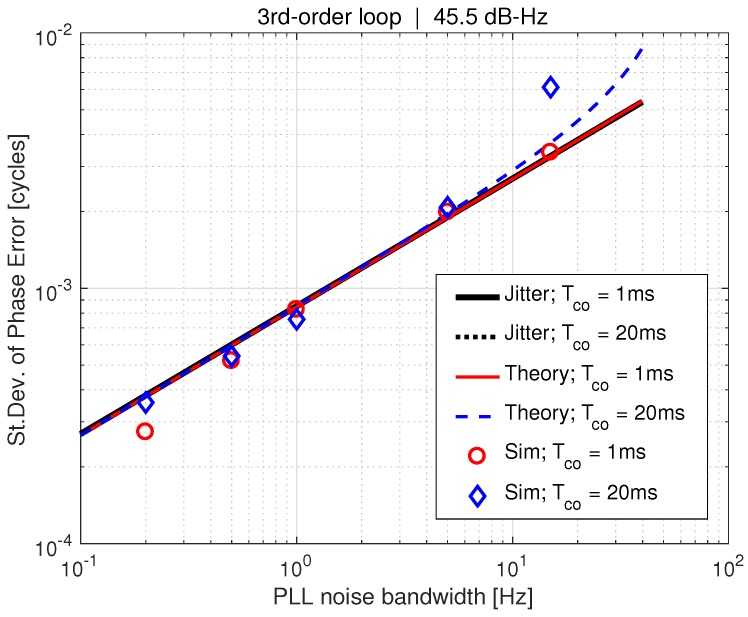
Sigma phase error vs. PLL bandwidth.

Figure 9 shows the same curves as Figure 8, except with the phase error standard deviation, $\sigma_{\phi }$, on the y-axis. In contrast to the previous results, $\sigma_{\phi }$ is a strong function of PLL bandwidth. On the other hand, coherent averaging time produces little effect (ignoring the bandwidth phenomenon associated with a 20 ms coherent averaging time). Additionally, the thermal noise term from the jitter metric (Equation (39)) is plotted for 1 ms and 20 ms averaging times. Note that the jitter metric curves coincide with our theoretical calculation using a 1 ms averaging time.

Figure 8 and Figure 9 have validated the augmented PLL linear model derived in Section 3, and confirmed a non-obvious effect indicated from our study of the theory (Section 6); tightening bandwidth does reject noise on the phase error, but has no effect on the tracking error. This turns out to be an important issue, as we will discuss next.

## 8. Tracking Error as a Design Metric

In this section, we take a closer look at phase error and tracking error results by plotting $\sigma_{\phi }$ vs. $\sigma_{\epsilon }$. For the AWGN only case (as we have considered thus far) $\sigma_{\phi }$ is equivalent to the jitter metric, as shown in Figure 9. Thus, we are essentially plotting the jitter metric versus our proposed metric, the standard deviation of tracking error.

**Figure 10:**
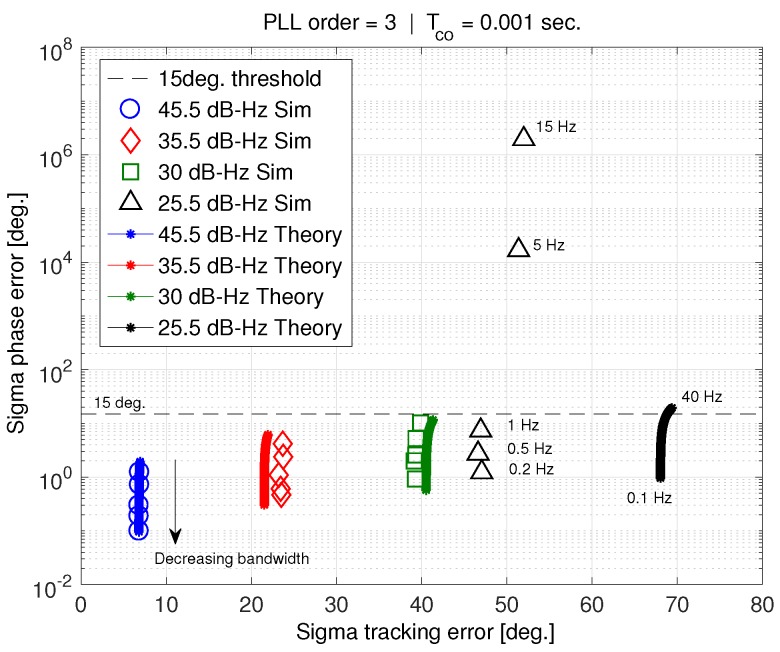
Sigma phase error vs. sigma tracking error.

Figure 10 shows sigma phase error versus sigma tracking error for a 1 ms coherent averaging time. Both theory and simulation results are presented. Each curve represents a constant carrier-to-noise ratio, and varying PLL bandwidth. Note that reducing PLL bandwidth reduces the sigma phase error, $\sigma_{\phi }$, but has little observable effect on the tracking error. This means that, in Figure 10, decreasing $\sigma_{\phi }$ corresponds to decreasing bandwidth. Bandwidth for the theory ranges between 40 Hz and 0.1 Hz, while PLL simulation is conducted for 30 s at bandwidths of 15, 5, 1, 0.5, and 0.2 Hz. The 15 degrees threshold on $\sigma_{\phi }$, corresponds to the standard jitter metric threshold [12]. Both theory and simulation results agree well until the standard deviation of tracking error, $\sigma_{\epsilon }$, reaches about 40 degrees. For larger $\sigma_{\epsilon }$, theory and simulation do not match in either $\sigma_{\phi }$ or $\sigma_{\epsilon }$.

The solid black curve in Figure 10, corresponding to $C/N_{0}=25.5$ dB-Hz, shows that the jitter metric only exceeds the 15 degrees threshold for large PLL bandwidths approaching 40 Hz. It can be deduced (by re-plotting Figure 9 for a 25.5 dB-Hz carrier-to-noise ratio) that this corresponds to bandwidths larger than 24 Hz. The phase jitter metric suggests that a PLL using any of the bandwidths in our simulation, would be able to track a carrier-to-noise ratio of 25.5 dB-Hz. However, the simulation results show extremely large $\sigma_{\phi }$ that do not match theory, because the phase error is in fact non-stationary over the 30 s simulation, and the PLL has lost lock. In addition, the standard deviation of tracking error saturates at about 52 degrees, even though theory predicts it to be much larger. This phenomenon is explained in greater detail in Section 8.1.

**Figure 11:**
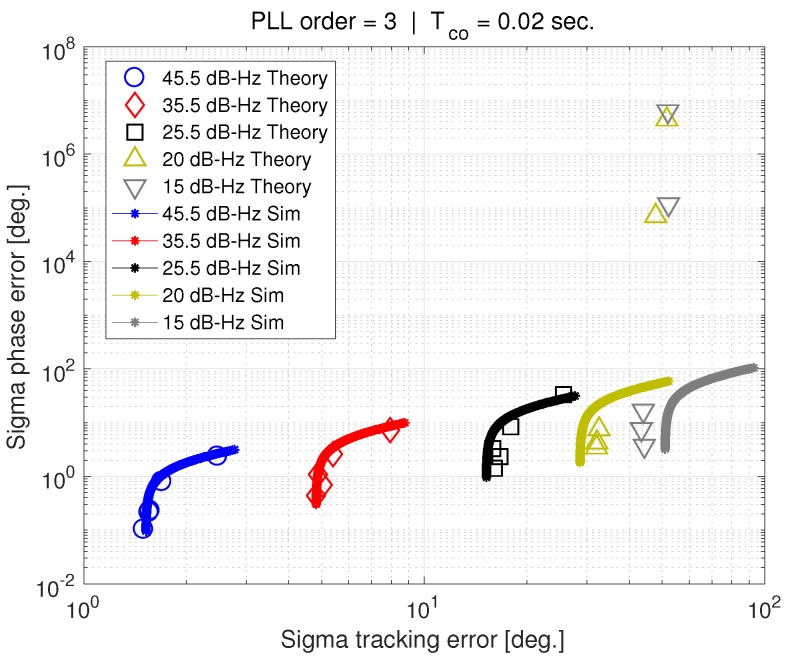
Sigma phase error vs. sigma tracking error, for $T_{co}$ = 20 ms.

Figure 11 shows $\sigma_{\phi }$ versus $\sigma_{\epsilon }$, but for a 20 ms coherent averaging time. In this case, the PLL is able to successfully track a 25.5 dB-Hz carrier-to-noise ratio. Theory and simulation agree well down to this noise level. According to [6], increasing the coherent average time should not make a difference to the $C/N_{0}$ that can be tracked. However, Figure 10 and Figure 11 show the opposite. Furthermore, the phase error standard deviation deviates from theory when the tracking error standard deviation approaches its saturation level of 52 degrees.

The theoretical computation of the standard deviation of phase error breaks down as $C/N_{0}$ decreases. However, the point at which this happens is dependent on factors such as the coherent averaging time. Figure 10 and Figure 11 illustrate what has been observed in previous work [4,5,6,7]. The phase jitter metric does not accurately predict cycle slipping and loss of lock in the presence of wideband interference. Our results suggest that the reason why is related to the tracking error standard deviation becoming saturated at 52 degrees. We believe this saturation is the result of the tracking error is exceeding the discriminator phase pull-in region (90 degrees for the arctangent discriminator) too frequently. This idea is investigated in greater depth next.

### 8.1. Probability Distribution of Tracking Error

To investigate the role of the discriminator in carrier tracking, the probability distribution of the tracking error is determined analytically and examined for varying carrier-to-noise ratios.

Equation (18) is used to derive the probability distribution of the tracking error. In this exercise, we assume the phase error ϕ is constant in time. Therefore, only $n_{I}^{\prime }\left(t\right)$ and $n_{Q}^{\prime }\left(t\right)$ are random variables. Equation (18) can be written as,

(43) $$\begin{array}{c} \epsilon \left(t\right)=\tan^{-1}\left(\frac{\phi +\langle n_{Q}^{\prime }\left(t\right)\rangle }{1+\langle n_{I}^{\prime }\left(t\right)\rangle }\right) \end{array}$$

with the coherent averaging only affecting the additive noise terms. The argument of the arctangent becomes a ratio of two Gaussian random variables with non-zero means,

(44) $$\begin{array}{cc} Z & =\tan^{-1}\left(\frac{Y}{X}\right) \end{array}$$

with, $Y∼N(\phi \;,\;\sigma_{n_{Q}^{\prime }}^{2}/N)$ and $X∼N(1\;,\;\sigma_{n_{I}^{\prime }}^{2}/N)$, where *N* is the number of samples being averaged. Random variables *X* and *Y* have the same variance ($\sigma_{n_{I}^{\prime }}^{2}=\sigma_{n_{Q}^{\prime }}^{2}$), which is proportional to the carrier-to-noise ratio. The distribution of *Z* is derived in Appendix C using a result from [21], and is non-zero between ±90 degrees.

**Figure 12:**
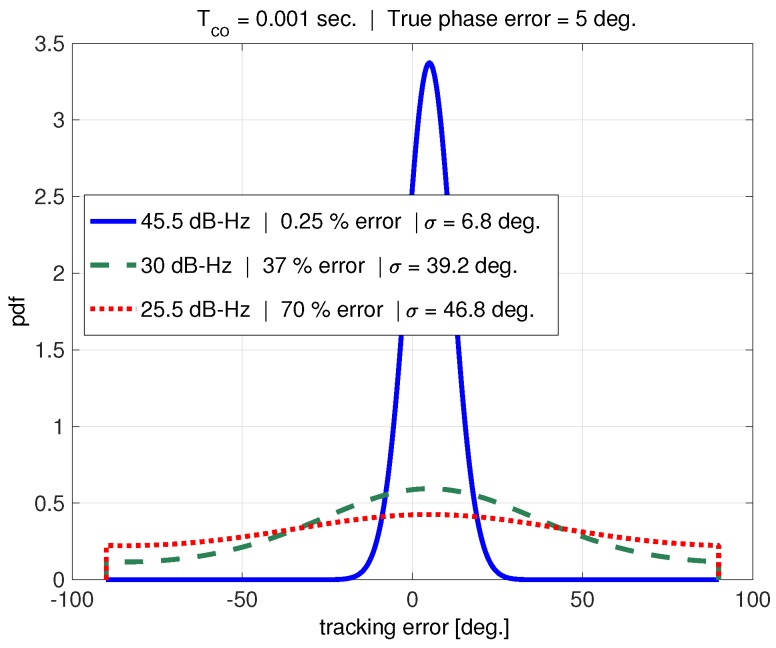
PDF of tracking error.

Figure 12 shows the probability distribution of the tracking error, ϵ, for a 1 ms coherent averaging time and (an example) true phase error, ϕ, of 5 degrees. The distributions corresponding to three different carrier-to-noise ratios are plotted. The figure legend includes the error with respect to the true phase error of five degrees (using the expected value to estimate the phase error), as well as the standard deviation of the distribution.

In the absence of interference, for example, a carrier-to-noise ratio of 45.5 dB-Hz, the tracking error has a very Gaussian-like distribution. The true phase error can be accurately estimated from this distribution using the expected value. As the carrier-to-noise ratio is decreased, the tracking error distribution approaches a uniform distribution. This makes it increasingly difficult for the loop filter (modeled by the expected value operator in this example) to obtain a reasonable estimate of the true phase error. The standard deviation of a uniform distribution over ±90 degrees is 52 degrees, which matches with the maximum $\sigma_{\epsilon }$ seen from simulation in Figure 10.

Examination of Figure 10, Figure 11 and Figure 12 suggests that the discriminator pull-in region plays an important role in cycle-slipping, and that the jitter metric does not adequately consider this effect.

While the jitter metric threshold is set based on the discriminator’s pull-in region, the metric does not account for the effect of the AWGN (see Figure 2) on the discriminator. As wideband interference levels increase, and $C/N_{0}$ decreases, the AWGN overwhelms the discriminator and the tracking error distribution approaches a uniform distribution as shown in Figure 12.

This is likely the reason why the phase jitter metric is not a trustworthy design metric. Our proposed metric based on the tracking error uses a linear model that includes coherent averaging. In addition, the threshold for this new metric is based upon the discriminator’s characteristics. Therefore, both coherent averaging as well as the discriminator are accounted for in the tracking error metric.

### 8.2. PLL Performance Metric Based on Tracking Error

The general form of the proposed tracking error performance metric is,

(45) $$\begin{array}{c} k\sigma_{\epsilon }+\epsilon_{D}\leq R \end{array}$$

where *k* is an inflation factor, $\epsilon_{D}$ is worst case dynamic stress (from all sources), and *R* is the discriminator pull-in region (90 degrees for the arctangent discriminator). The dynamic stresses can be accounted for in the same way as in the phase jitter metric. The worst case tracking error can be approximated by the steady state value of tracking error due to a particular stressor. Practical values of the inflation factor will be, k≥2.

For example, with k=2 and no dynamic stress, the metric becomes $\sigma_{\epsilon }\leq 45$ degrees. In other words, two-sigma tracking error must not exceed the discriminator’s pull-in region. The tracking error distribution for this case roughly corresponds to the 25.5 dB-Hz curve in Figure 12. The distribution is approaching a uniform distribution, so the mean-time to cycle slip (MTCS) is likely not very long. This case can be used as a quick feasibility check when designing PLLs. In practice, *k* should be inflated to reduce the maximum tolerable tracking error standard deviation. This will yield a more significant MTCS by ensuring that the tracking error distribution does not become uniform.

The results in this section have validated the augmented linear model (developed in Section 3) using simulation up until a 52 degree standard deviation of tracking error, $\sigma_{\epsilon }$. This threshold was shown to be significant in that it is representative of the tracking error distribution being uniformly distributed over the discriminator’s pull-in region. The tracking error PLL performance metric was defined to guarantee the tracking error distribution does not approach a uniform distribution.

The standard deviation of tracking error, $\sigma_{\epsilon }$, has the potential to be much more reliable as a PLL design metric than the phase jitter metric, $\sigma_{PLL}$ (which is equivalent to $\sigma_{\phi }$ for the AWGN only case). However, in a realistic scenario, the reference oscillator phase-noise will impact the tracking error and must also be accurately modeled.

## 9. Experimental Results and Validation

To validate the theoretical results, including clock phase-noise effects, experimental data are collected using a signal generator and receiver front-end, and processed with a software defined receiver (SDR).

A Spectracom GSG-6 series signal generator with a SpectraTime LPFRS-01/AV1 rubidium reference oscillator is used to simulate GPS satellite signals. A USRP N200 with DBSRX2 daughterboard and separate LPFRS reference oscillator is used as the GPS receiver front end. The front-end outputs an IF signal at 5 MHz with a sampling frequency of 20 MHz which is saved for post-processing. Finally, a software defined receiver (SDR) based on that provided with [14] is used to acquire and track the signal. The signal is acquired with a 45.5 dB-Hz carrier-to-noise ratio, which is decreased to the desired level once PLL tracking is initiated to simulate a broadband interference event.

**Figure 13:**
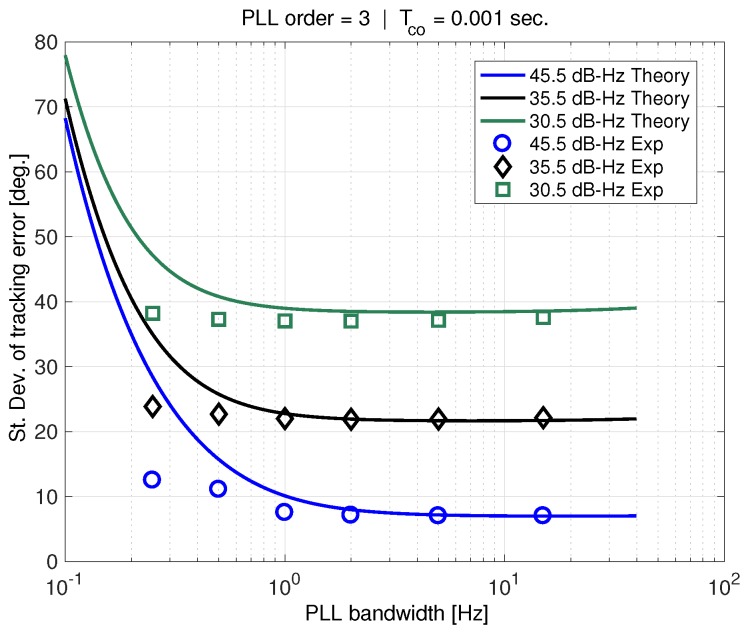
Experimental results with theory using Rb PSD model.

Figure 13 shows experimental tracking error results for varying carrier-to-noise ratios compared to theory which now includes the effect of receiver clock phase-noise. The phase-noise PSD corresponds to the LPFRS oscillator actually used. The theory is represented using solid curves while experimental results are plotted using markers. There is very good agreement between theory and experiment for PLL bandwidths greater than about 1 Hz. (Note that the AWGN and clock phase-noise add directly in the new linear model. Thus, generally for high PLL bandwidths, the the additive white noise dominates.) However, at lower bandwidths where the clock phase-noise model dominates, there is disagreement. The PSD power-law model for the LPFRS oscillator computed using the method described in Appendix A appears to be overly conservative.

**Figure 14:**
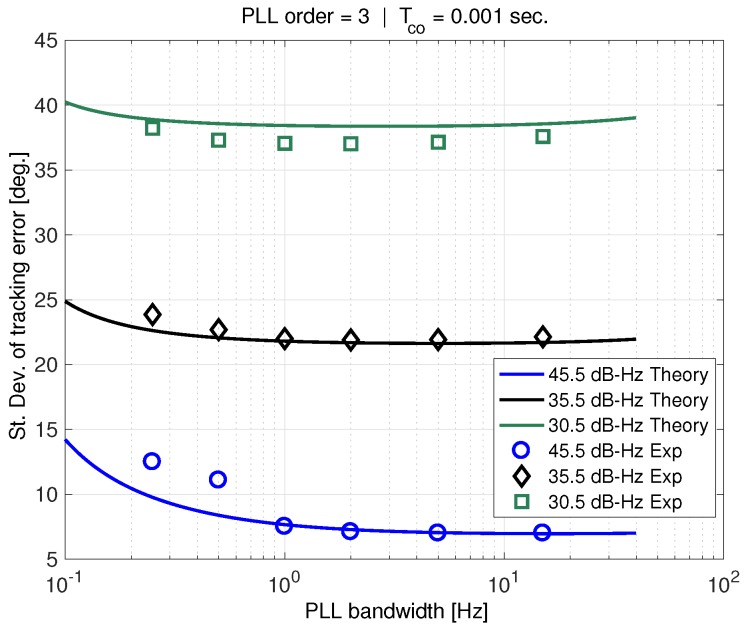
Experimental results with theory using OCXO PSD model.

Figure 14 shows the same experimental results compared to theory utilizing the OCXO clock model PSD from Table 1. This phase-noise model is a much better approximation to that of the actual oscillator. There is still a mismatch at low PLL bandwidths for $C/N_{0}=45$ dB-Hz, but the correspondence is better for lower carrier-to-noise ratios. At 30.5 dB-Hz, the AWGN essentially dominates over the entire range of PLL bandwidths.

Accurately modeling oscillator phase-noise is necessary to determine the extent to which the PLL bandwidth can be tightened. As shown in Section 7, this improves the PLL output quality, and therefore will improve the quality of carrier-phase measurements. A low phase-noise clock is desirable because it does not intensify the standard deviation of tracking error as much as a clock with higher phase-noise, and can tolerate more interference at lower PLL bandwidths before the tracking error threshold is reached.

## 10. Conclusions

In this work, we derived an augmented GPS phase-lock loop (PLL) linear model which includes the effect of coherent averaging. It was shown that the augmented model allows for accurate calculation of tracking error standard deviation in the presence of additive white Gaussian noise (AWGN) and clock phase-noise, given an accurate input clock phase-noise PSD model.

The standard deviation of tracking error was shown to be a useful PLL performance/design metric. Using both PLL simulation and experiment, a threshold of 45 degrees on the tracking error standard deviation was shown to be a more reliable/robust indicator of PLL performance under interference conditions than the widely used phase jitter metric. In addition, the augmented linear model is shown to be valid up until this threshold, which facilitates efficient performance prediction, without the need for time-consuming direct PLL simulation.

This new performance metric can be used to evaluate the performance of stationary GPS receivers that are vulnerable to wideband radio frequency interference (RFI). The method is directly applicable to ground-based reference receivers for differential GPS systems such as the Ground Based and Space Based Augmentation Systems (GBAS and SBAS), as well as other ground-based receivers that require high continuity of service. It is also relevant to roving receivers, if the additional dynamic stresses on the PLL are also taken into account.

Recommendations based on the aforementioned results would be to maximize coherent averaging time, which has the most significant effect in reducing the PLL tracking error standard deviation. This should be the priority when the goal is maintaining lock (i.e., preventing cycle slips). Using an oscillator with low phase-noise is also important because it will allow the receiver to handle more AWGN. In addition, it will allow the PLL bandwidth to the tightened, improving PLL stability and carrier measurement quality.

## Figures and Tables

**Table 1:** Clock model power spectral density (PSD) coefficients.

d	TCXO	OCXO	Rubidium
$h_{0}$	$5.0\times 10^{-8}$	$5.5\times 10^{-8}$	$5.0\times 10^{-8}$
$h_{1}$	$6.2\times 10^{-5}$	$5.0\times 10^{-5}$	$6.2\times 10^{-5}$
$h_{2}$	$9.6\times 10^{-4}$	$6.5\times 10^{-4}$	$5.3\times 10^{-4}$
$h_{3}$	$6.0\times 10^{-3}$	$9.0\times 10^{-7}$	$6.9\times 10^{-4}$
$h_{4}$	$6.0\times 10^{-4}$	$1.0\times 10^{-7}$	$1.2\times 10^{-10}$

**Figure A1:**
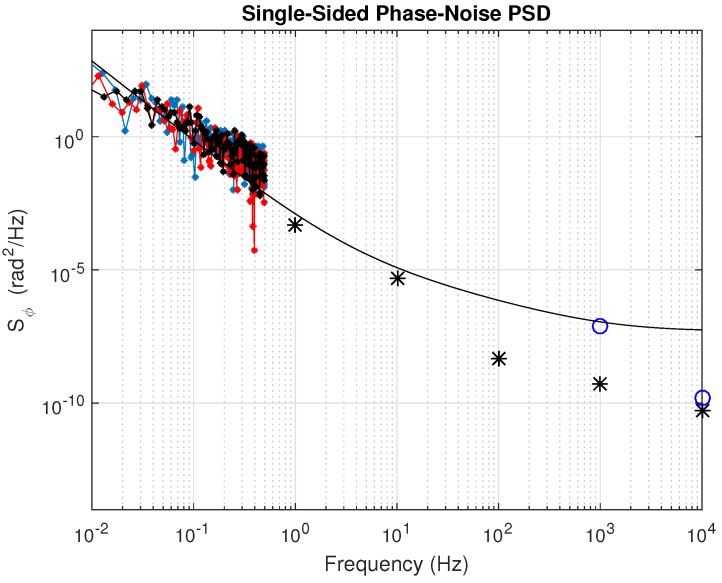
LPFRS measurements and specs with fitted power-law model.

## Appendix A. LPFRS-01 Power-Law Model

In the experimental part of this work, a SpectraTime LPFRS-01/AV1 rubidium oscillator was used as the receiver reference clock. To obtain the most accurate prediction of PLL performance, a phase noise power spectral density (PSD) power-law model representative of the particular oscillator we are using was required. The generic rubidium model from [5] is likely not accurate enough.

The manufacturer’s phase-noise PSD specifications for the LPFRS were augmented with our own measurements to form the power-law model. For the latter, we followed the method used by Alban [22] ([23], p. 120–123). Velocity measurements are used to determine the reference clock PSD at low offset frequencies, where PSD specifications are not provided by the manufacturer.

It is important to note that Alban’s method of using velocity measurements includes the effects of frequency synthesis. We believe the generic rubidium power-law model in prior work [5] does not consider the effects of frequency synthesis.

Measurements were taken using a Novatel DL-v3 receiver capable of using an external reference oscillator. Clock bias rate data were collected directly (rather than computed from velocity measurements) using the receiver’s “clockmodel” log. See [22] ([23], p. 120–123) for additional information on how the power-law model is formed.

Figure A1 shows the resulting phase noise PSD for the LPFRS-01 rubidium oscillator, including the effects of frequency synthesis. The worst case is considered, where the frequency synthesis generates L1 (mixing to baseband), which results in the highest phase-noise on synthesizer output.

## Appendix B. Transfer Function for Moving Average

Begin with the average of *N* samples,

(A1) $$\begin{array}{c} y_{k}=\frac{1}{N}\left(x_{k}+x_{k-1}+\cdots +x_{k-(N-1)}\right) \end{array}$$

Taking the Z-transform,

(A2) $$\begin{array}{cc} \frac{y_{k}}{x_{k}} & =\frac{1}{N}\left(1+z^{-1}+\cdots +z^{-(N-1)}\right)\\ & =\frac{1}{N}\sum_{p=0}^{N-1}z^{-p}\\ & =\frac{1}{N}\left(\frac{z^{N-1}}{z^{N-1}}\right)\sum_{p=0}^{N-1}z^{-p}\\ & =\frac{1}{N}\left(\frac{1}{z^{N-1}}\right)\sum_{q=0}^{N-1}z^{q} \end{array}$$

This series has a solution of the form,

(A3) $$\begin{array}{c} \sum_{k=0}^{n}z^{k}=\frac{1-z^{n+1}}{1-z} \end{array}$$

Thus, we have,

(A4) $$\begin{array}{cc} \frac{y_{k}}{x_{k}} & =\frac{1}{N}\left(\frac{1}{z^{N-1}}\right)\frac{1-z^{N}}{1-z}\\ & =\frac{1}{N}\frac{z^{-(N-1)}-z}{1-z}\\ & =\frac{1}{N}\frac{z-z^{1-N}}{z-1}\\ \frac{y_{k}}{x_{k}} & =\frac{1}{N}\frac{1-z^{-N}}{1-z^{-1}} \end{array}$$

Now, to convert to continuous, we have $z\to e^{sT}$, where *T* is the sampling period.

(A5) $$\begin{array}{cc} C(s) & =\frac{1}{N}\frac{1-e^{-sTN}}{1-e^{-sT}} \end{array}$$

Furthermore, we can write this equation using the coherent averaging time, $T_{co}=NT$,

(A6) $$\begin{array}{cc} C(j\omega ) & =\frac{T}{T_{co}}\frac{1-e^{-j\omega T_{co}}}{1-e^{-j\omega T}} \end{array}$$

## Appendix C. Tracking Error Distribution

The ratio in Equation (44) is defined as random variable *W*,

(A7) $$\begin{array}{c} W:=\frac{Y}{X} \end{array}$$

where, $Y∼N(\mu_{Y}\;,\;\sigma_{Y}^{2})$ and $X∼N(\mu_{X}\;,\;\sigma_{X}^{2})$. Here, $\mu_{Y}=\phi$ (where ϕ is assumed to be a known constant), $\mu_{X}=1$, and $\sigma_{X}^{2}=\sigma_{Y}^{2}=\sigma_{n_{Q}^{\prime }}^{2}/N$.

The distribution of *W*, denoted $f_{W}\left(w\right)$, is given in [21] and repeated here for convenience.

(A8) $$\begin{array}{cc} f_{W}\left(w\right)= & \frac{b(w)d(w)}{\sqrt{2\pi }\sigma_{X}\sigma_{Y}a^{3}\left(w\right)}\left[\Phi \left(\frac{b(w)}{a(w)}\right)-\Phi \left(-\frac{b(w)}{a(w)}\right)\right]\\ & +\frac{1}{a^{2}\left(w\right)\pi \sigma_{X}\sigma_{Y}}e^{-c(w)/2} \end{array}$$

where Φ is the Gaussian cumulative distribution function, and,

(A9) $$\begin{array}{ll} a(w) & =\sqrt{\frac{w^{2}}{\sigma_{Y}^{2}}+\frac{1}{\sigma_{X}^{2}}} \end{array}$$

(A10) $$\begin{array}{ll} b(w) & =\frac{\mu_{Y}}{\sigma_{Y}^{2}}w+\frac{\mu_{X}}{\sigma_{X}^{2}} \end{array}$$

(A11) $$\;\begin{array}{ll} c(w) & =\frac{\mu_{Y}^{2}}{\sigma_{Y}^{2}}+\frac{\mu_{X}^{2}}{\sigma_{X}^{2}} \end{array}$$

(A12) $$\;\begin{array}{ll} d(w) & =\exp\left[\frac{b{\left(w\right)}^{2}-c\left(w\right)a^{2}\left(w\right)}{2a^{2}\left(w\right)}\right] \end{array}$$

The distribution of $Z:=\tan^{-1}\left(W\right)$ (equivalent to definition of *Z* in Equation (44)) may be computed as,

(A13) $$\begin{array}{cc} f_{Z}\left(z\right) & =\frac{1}{\left|dz/dw\right|}f_{W}\left(\tan\left(z\right)\right) \end{array}$$

(A14) $$\begin{array}{cc} f_{Z}\left(z\right) & =\sec^{2}\left(z\right)\;f_{W}\left(\tan\left(z\right)\right) \end{array}$$

where *z* is between ±π/2 (otherwise $f_{Z}\left(z\right)=0$).
